# Effect of New Zealand Blackcurrant Extract on Performance during the Running Based Anaerobic Sprint Test in Trained Youth and Recreationally Active Male Football Players

**DOI:** 10.3390/sports5030069

**Published:** 2017-09-15

**Authors:** Charlie Godwin, Matthew D. Cook, Mark E. T. Willems

**Affiliations:** 1 Department of Sport and Exercise Sciences, University of Chichester, College Lane, Chichester, West Sussex PO19 6PE, UK; charliegod15@hotmail.co.uk; 2 Institute of Sport and Exercise Science, University of Worcester, Henwick Grove, Worcester WR2 6AJ, UK; matthew.cook@worc.ac.uk

**Keywords:** football, running sprints, fatigue, elite athletes, anthocyanins, polyphenols

## Abstract

It was observed previously that New Zealand blackcurrant (NZBC) extract reduced slowing of the maximal 15 m sprint speed during the Loughborough Intermittent Shuttle Test. We examined the effect of NZBC extract on the performance of the Running Based Anaerobic Sprint Test (RAST, 6 × 35-m sprints with 10 seconds passive recovery) in trained youth and recreationally active football players. Fifteen recreationally active (University team) (age: 20 ± 1 years, height: 174 ± 19 cm, body mass: 80 ± 13 kg) and nine trained youth players (English professional club) (age: 17 ± 0 years, height: 178 ± 8 cm, body mass: 69 ± 9 kg, mean ± SD) participated in three testing sessions. Prior to the RASTs, participants consumed two capsules of NZBC extract (600 mg∙day^−1^ CurraNZ^®^) or placebo (P) for 7 days (double blind, randomised, cross-over design, wash-out at least 14 days). Ability difference between groups was shown by sprint 1 time. In the placebo condition, trained youth players had faster times for sprint 1 (5.00 ± 0.05 s) than recreationally active players (5.42 ± 0.08 s) (*p* < 0.01). In trained youth players, there was a trend for an effect of NZBC extract (*p* = 0.10) on the slowing of the sprint 1 time. NZBC extract reduced slowing of the sprint 5 time (P: 0.56 ± 0.22 s; NZBC: 0.35 ± 0.25, *p* = 0.02) and this was not observed in recreationally active players (P: 0.57 ± 0.48 s; NZBC: 0.56 ± 0.33, *p* = 0.90). For fatigue index, expressed as a % change in fastest sprint time, there was a strong trend to be lower in both trained youth and recreationally active players combined by NZBC extract (P: −13 ± 7%; NZBC: −11 ± 6%, *p* = 0.06) with 12 participants (five trained youth) experiencing less fatigue. New Zealand blackcurrant extract seems to benefit repeated sprint performance only in trained football players.

## 1. Introduction

Blackcurrant contains primarily the anthocyanins delphinidin-3-glucoside, delphinidin-3-rutinoside, cyanidin-3-glucoside and cyanidin-3-rutinoside [1]. Anthocyanins contribute to a wide range of human health benefits including suppression of proliferation in tumor cell lines [2], improved LDL cholesterol in diseased patients [3], improved vision in patients with normal tension glaucoma [4], increased cognitive function in healthy young adults [5] and may enhance exercise-induced immune function [6]. Anthocyanins have also been associated with anti-inflammatory [7] and antioxidant activity [6,8] and may therefore counteract negative physiological effects that may occur during (e.g., fatigue) and after exercise (e.g., inflammation).

Anthocyanins act on the vascular endothelium [9] and increase endothelial nitric oxide synthase activity with production of nitric oxide to vasodilate blood vessels in skeletal muscle [10]. Therefore, anthocyanins increase peripheral blood flow during exercise. For example, Matsumoto et al. [11] observed that peripheral forearm blood flow increased by 22% and reduced fatigue during typing work in humans three hours after blackcurrant intake. Furthermore, intake of blackcurrant extract increased the femoral artery diameter during submaximal sustained isometric exercise [12]. In general, an increase in blood flow during exercise may enhance performance.

Cook et al. [13] observed in trained cyclists an enhanced performance by 2.4% for a 16.1 km cycling time trial. Beneficial effects of blackcurrant intake on performance were also observed during repeated high-intensity running exercise. For example, Perkins et al. [14] observed that New Zealand blackcurrant intake increased repeated high-intensity treadmill running by 10.8%. In the study by Perkins et al. [14], the treadmill running protocol that was used correlated with the YoYo IR2 test reported by Krustrup et al. [15]. In addition, better maintenance of maximal sprinting ability was also observed during the Loughborough Intermittent Shuttle Test [16]. Therefore, it seems that blackcurrant may be ergogenic for activities that are typically part of team sports such as football. Performance analysis has revealed that sprinting constitutes approximately 10% of the total distance covered during a football match [17]. In recent years, there has been an 80% increase in the number of sprints performed in the premier league [18]. The ability to perform repeated sprints has been associated with football match-related physical performance [19] and is usually assessed by coaches and applied sports scientists in the field.

The ability to repeatedly perform maximal sprints (≤10 s) with short recovery periods (≤10) has been termed repeated sprint ability [20]. The running anaerobic sprint test has been popular to quantify anaerobic power and fatigue in a non-laboratory testing environment [21]. The test is highly reliable and correlates with the Wingate test (peak power *r* = 0.46; mean power *r* = 0.53; fatigue index *r* = 0.63) and 35, 50, 100, 200 and 400 m sprint performance [22]. Ergogenic effects of New Zealand blackcurrant on maximal repeated sprint ability during the running anaerobic sprint test are not known. 

Recently, some studies have examined whether training status affects the ergogenic properties of performance-enhancing supplementation. For caffeine, it was reported that trained subjects only experienced beneficial effects during morning testing. However, for beta-alanine, the ergogenic effects were beneficial for untrained and trained cyclists for lower-body Wingate tests [23]. It is not known whether a potential performance enhancing effect by New Zealand blackcurrant extract for repeated maximal running sprints depends on training status.

Therefore, the aims of the present study were to examine the effects of 7-day intake of a New Zealand blackcurrant extract on the performance during the running anaerobic sprint test and whether trained youth and recreationally active football players would show different performance responses.

## 2. Materials and Methods

### 2.1. Participants

Nine healthy trained youth and fifteen recreationally male football players volunteered. After explanation of the experimental protocol and procedures, potential risks and benefits, participants completed a health history questionnaire and provided written informed consent. Trained youth football players were recruited from a team of an English professional club. Recreationally active football players were recruited from a University team. Approval for the study was granted by the University of Chichester Research Ethics Committee (Code: 1516_39) with experimental protocols and procedures conforming to the 2013 Declaration of Helsinki. Participants did not receive payment for participation.

### 2.2. Experimental Design

The study consisted of three testing sessions within five weeks. In the first visit, participants performed a multistage fitness test to exhaustion to establish predicted $\dot{V}$O_2max_ (maximum oxygen uptake). After a 20-min rest, participants were familiarised with the running anaerobic sprint test. Following familiarisation, participants were randomly assigned in a double-blind, placebo controlled, cross-over design and consumed two capsules of New Zealand blackcurrant extract or identical looking placebo capsules every morning with breakfast for 7 days. On the final day of supplementation, participants performed testing at the same time in the morning to limit any circadian rhythm variation and approximately 2 h after breakfast. Testing sessions two and three were separated by a period of at least 21 days which allowed a minimum 14-day washout period and a second supplementation period of 7 days.

### 2.3. Anthropometry Characteristics

Anthropometric parameters were recorded in the first testing session. Body height and body mass were measured using a portable stadiometer (Leicester Height Measure MKII, Invicta Plastics, Leicester, UK) and digital scales (Seca Model 876, Seca Ltd, Birmingham, UK). To estimate adiposity, skinfold thickness was measured at four sites on the right side of the body (i.e., biceps, triceps, subscapular and suprailiac skinfolds) [24]. Skinfold thickness was measured using a Harpenden skinfold calliper (Bodycare products, Southam, Warwickshire, UK) to the nearest 0.2 mm in accordance with the International Society for the Advancement of Kinanthropometry (ISAK). Sum of skinfolds and body fat percentage were calculated using the equations described by Durnin and Womersley [24].

### 2.4. Dietary Standardisation

Each participant recorded their dietary intake in a written food diary for the 48 h prior to the first running anaerobic sprint test and were instructed to replicate the dietary intake before the second running anaerobic sprint test. Participants were instructed not to perform vigorous exercise, not to consume alcohol 48 h before each testing session, be well-rested and hydrated on arrival. Participants were also instructed not to consume other supplementation during the study that may add further nutritional value to their diet.

### 2.5. Independent Physical Activity Questionnaire

Participants were instructed to keep their weekly exercise schedule as consistent as possible throughout the study. Prior to the first running anaerobic sprint test, each participant completed an international physical activity questionnaire to determine the total amount of physical activity performed for the previous 7 days. Specifically, the international physical activity questionnaire assessed physical activity performed across a comprehensive set of domains including leisure time, domestic gardening, work-related and transport-related activity. The total metabolic equivalent for the 7-day period was calculated.

### 2.6. Anthocyanin Content

Prior to the first running anaerobic sprint test, each participant completed a food frequency questionnaire specifying an average consumption for each fruit, vegetable and drink during the past year (i.e., never, less than 1 per month, 1 per month, 2–3 per month, once a week, 3–4 per week, 5–6 per week or every day). In addition, each participant selected a typical serving size (i.e., small, medium or large). The total daily anthocyanin intake amount for each fruit, vegetable and drink could be calculated individually using the following equation: serving size × anthocyanin content of fruit, vegetable or drink, using Phenol Explorer (http://phenol-explorer.eu/). Once calculated, the total amount of anthocyanins consumed during the past year was determined by adding together the total daily anthocyanin intake for each fruit, vegetable and drink. The estimated daily anthocyanin intake is presented in Table 1.

### 2.7. Estimation of Maximal Oxygen Uptake 

Participants performed a 20-m multistage fitness test to exhaustion to establish predicted $\dot{V}$O_2max_. The multistage fitness test required participants to perform shuttle runs back and forth between two lines 20-m apart at a progressively increased speed controlled by an audio signal from a CD player. The test began at an initial running velocity of 8.5 km·hr^−1^ and increased incrementally by 1 km·hr^−1^ each level. When the participant failed twice to reach the finishing line in time or could no longer run at the imposed running velocity, heart rate (Polar Heart Rate Monitors, F1, Polar Electro UK Ltd., Warwick, UK), total distance, and the final stage completed were recorded. Participants ran in groups of no more than four to ensure maximal effort. Verbal encouragement was provided during the test. Predicted $\dot{V}$O_2max_ for each participant was calculated using an equation described by Leger et al. [25]. Participant characteristics are presented in Table 1.

### 2.8. Sprint Criterion Threshold

Prior to establishment of the sprint criterion threshold in each sprint session, participants performed a standardised warm up consisting of dynamic stretches and submaximal intensity running. Following a 2-min rest period, each participant performed a 35-m maximal individual sprint to determine their sprint criterion threshold. The sprint criterion threshold was recorded to prevent a pacing strategy during the running anaerobic sprint test [26]. Once the sprint criterion threshold was determined, participants were instructed to have a 5-min recovery period before starting the running anaerobic sprint test. During the first sprint of the running anaerobic sprint test, participants were required to achieve at least 95% of their sprint criterion threshold. If the time of the first sprint time was less than 95% of the individuals’ sprint criterion threshold, participants were instructed to have a 5-min recovery period and recommence the running anaerobic sprint test. However, every participant achieved at least 95% of their sprint criterion threshold during the first sprint.

### 2.9. Running Anaerobic Sprint Test (RAST)

The running anaerobic sprint test was performed on an artificial football pitch for the trained youth football players and University athletics track for the recreationally active football players. Environmental conditions were recorded (environmental temperature: 11 ± 1 °C; humidity: 65 ± 3%; air pressure: 1015 ± 4 mbar, wind speed 7 ± 4 km·h^−1^). The test consisted of 6 × 35-m maximal sprints interspersed by 10-second passive recovery periods. Each sprint was recorded using a photocell system (Fusion Smartspeed lightgates, Hab International, Warwickshire, UK) which were positioned on the start line and finish line at a height of 75 cm. Each sprint began from a standing start position. The standing start position was adopted 30 cm behind the start line to avoid breaking the infrared beam produced by the photocell system. Sprint time was recorded and fatigue index (FI) calculated using the following equation.

(1) $$Fatigue\;index\;\left(\%\right)=\frac{fastest\;sprint\;time-slowest\;sprint\;time}{fastest\;sprint\;time}\;\times \;100\%$$

After each sprint, the rating of perceived exertion (RPE) [27] was recorded.

### 2.10. Supplementation

Prior to the running anaerobic sprint test, participants consumed two capsules of concentrated New Zealand blackcurrant extract (one capsule of 300 mg contains 35–50% delphinidin-3-rutinoside, 5–20% delphinidin-3-glucoside, 30–45% cyanidin-3-rutinoside, and 3–10% cyanidin-3-glucoside, CurraNZ^®^, Health Currancy Ltd, Surrey, UK) or two identical looking placebo capsules (one capsule contains 300 mg microcrystalline cellulose M102, Lifesource Supplements, Ripon, UK) every morning with breakfast for 7 days.

### 2.11. Statistical Analysis

Statistical analyses were conducted on SPSS version 20.0 for Windows (SPSS Inc., Chicago, IL, USA). Data normality assumptions were assessed using the Kolmogorov–Smirnov test. For the data of both trained youth and recreationally active football players, a two-way repeated-measures ANOVA (analysis of variance) was conducted to analyse differences between the supplement conditions (New Zealand blackcurrant vs. placebo) by sprint time and RPE during the running anaerobic sprint test. Within each group, a two-way repeated-measures ANOVA was conducted to analyse the change in sprint time for sprints 2–6 between the placebo and blackcurrant condition with post-hoc *t*-tests when appropriate. Mauchly’s test of sphericity was conducted to test for homogeneity of data and if violated (*p* < 0.05), the Greenhouse-Geisser adjustment value was used. For both groups combined, the fatigue index was analysed with paired samples *t*-test. To determine the effect sizes, Cohen’s *d* was calculated [28]. Data were presented as mean (±SD) and statistical significance was set at an alpha level of *p* ≤ 0.05. Interpretation of 0.05 > *p* ≤ 0.1 was according to guidelines by Curran-Everett and Benos [29].

## 3. Results

### 3.1. New Zealand Blackcurrant vs. Placebo

In both conditions, for all participants combined, there was an increase in sprint time and RPE (*p* < 0.05) (Table 2) during the running anaerobic sprint test. However, there were no differences between conditions for sprint time (*p* = 0.25) and RPE (*p* = 0.63). In addition, there were no interaction effects between conditions for sprint time (*p* = 0.75) and RPE (*p* = 0.63).

**Figure 1:**
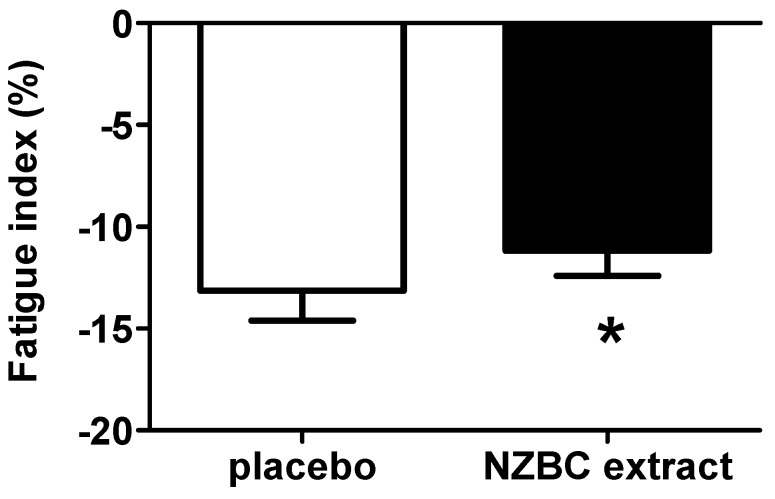
Fatigue index of the running anaerobic sprint test for trained youth and recreationally active football players for the placebo and New Zealand blackcurrant (NZBC) extract condition. * Denotes a strong trend for a difference between placebo and New Zealand blackcurrant extract (*p* = 0.06).

For both young trained and recreationally active football players, there was a strong trend for the fatigue index to be 12% lower following New Zealand blackcurrant extract compared to placebo (PLA: −13 ± 7 vs. NZBC: −11 ± 6%, range: −68%–26%, 12 participants showed a decrease and 12 no change) (*p* = 0.06) (*d* = −0.3) (Figure 1).

### 3.2. Sprint Performance

New Zealand blackcurrant extract had no effect on maximum sprint time (PLA: 5.61 ± 0.14 vs. NZBC: 5.54 ± 0.24 s, *p* = 0.35), minimum sprint time (PLA: 4.99 ± 0.14 vs. NZBC: 5.05 ± 0.30 s, *p* = 0.59) and mean sprint time (PLA: 5.34 ± 0.12 vs. NZBC: 5.29 ± 0.23 s, *p* =0.52) in trained youth football players. Similarly, New Zealand blackcurrant extract had no effect on maximum sprint time (PLA: 6.10 ± 0.42 vs. NZBC: 5.96 ± 0.41 s, *p* = 0.13), minimum sprint time (PLA: 5.38 ± 0.30 vs. NZBC: 5.33 ± 0.28 s, *p* = 0.39) and mean sprint time (PLA: 5.73 ± 0.27 vs. NZBC: 5.67 ± 0.32 s, *p* = 0.38) in recreationally active football players.

### 3.3. Change in Sprint Performance Compared to Baseline

Within each group separately, a two-way ANOVA was conducted to analyse the changes in sprint times that occurred during the RAST in placebo and NZBC conditions.

**Figure 2:**
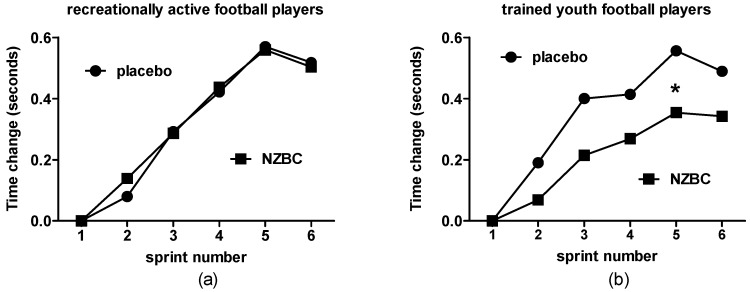
Change in sprint time from sprint 1 to subsequent sprints in recreationally active (**a**) and trained youth football players (**b**) following 7 days of New Zealand blackcurrant extract. * Denotes difference between placebo and New Zealand blackcurrant extract (*p* < 0.05). Data are mean values.

In recreationally active football players, New Zealand blackcurrant extract had no effect on the change in sprint times compared to the fastest sprint time of sprint 1 in sprint 2 (PLA: 0.08 ± 0.15 vs. NZBC: 0.14 ± 0.21 s, *p* = 0.37), sprint 3 (PLA: 0.29 ± 0.21 vs. NZBC: 0.29 ± 0.29, *p* = 0.94), sprint 4 (PLA: 0.42 ± 0.28 vs. NZBC: 0.44 ± 0.33, *p* = 0.84), sprint 5 (PLA: 0.57 ± 0.48 vs. NZBC: 0.56 ± 0.33, *p* = 0.90) and sprint 6 (PLA: 0.52 ± 0.36 vs. NZBC: 0.50 ± 0.38 s, *p* = 0.86) (Figure 2a).

In trained youth football players, there was a trend for a condition effect (*p* = 0.10). New Zealand blackcurrant extract reduced slowing of the fastest time of sprint 1 in sprint 5 (PLA: 0.56 ± 0.22 vs. NZBC: 0.34 ± 0.34 s, *p* = 0.02, *d* = −1) but not in sprint 2 (PLA: 0.19 ± 0.20 vs. NZBC: 0.07 ± 0.20 s, *p* = 0.15, sprint 3 (PLA: 0.40 ± 0.22 vs. NZBC: 0.21 ± 0.23 s, *p* = 0.16), sprint 4 (PLA: 0.41 ± 0.26 vs. NZBC: 0.27 ± 0.25 s, *p* = 0.29) and sprint 6 (PLA: 0.49 ± 0.23 vs. NZBC: 0.34 ± 0.34 s, *p* = 0.26) (Figure 2b).

## 4. Discussion

The main objective of the present study was to examine the effects of New Zealand blackcurrant extract on performance during the running based anaerobic sprint test in trained youth and recreationally active football players. For all participants combined (*n* = 24), 7-day intake of New Zealand blackcurrant extract reduced the fatigue index by 12%, with a high number of non-responders. Brocherie et al. [30] observed that the fatigue index during the RAST correlated with activity level of the *m.rectus femoris* and *m.biceps femoris*, but not with *m.vastus lateralis* in professional football players. Thus, it seems likely that the intake of New Zealand blackcurrant extract allows some football players to better maintain activity level in some lower limb muscles during repeated sprint running.

We also observed that the intake of New Zealand blackcurrant extract attenuated the increase in sprint time during sprint 5 only for the trained youth football players but not for the recreationally active football players. It is possible that our study was underpowered to show the effect at other sprints for the trained youth football players. Nevertheless, our observations seem to indicate that the intake of New Zealand blackcurrant was more effective for sprint performance in trained football players. Cumulative sprint times correlated with mean re-oxygenation rates in professional football players [30]. Thus, in the present study, some trained youth football players may have had better re-oxygenation rates during the RAST by intake of New Zealand blackcurrant extract. Intake of blackcurrant enhanced blood flow during typing [11] and submaximal sustained isometric exercise [12]. Enhanced blood flow during the brief recovery periods during the RAST may have benefitted sprint performance by allowing higher phosphocreatine resynthesis, and improved removal of metabolites that result from high energy turnover such as hydrogen ions, inorganic phosphate and adenosine diphosphate that can have a negative effect on the force production by the actomyosin cross-bridge cycle [31]. The mechanisms for muscle fatigue during repeated sprints are complex, and an effect on those mechanisms by blackcurrant intake seems to occur. However, in vitro studies on the effects of plasma metabolites that result from blackcurrant anthocyanin intake on peripheral muscle fatigue are warranted.

Whilst the effects of the New Zealand blackcurrant extract could remove unfavourable metabolites via anthocyanin induced endothelium dependant vasorelaxation [32], blackcurrant intake may also have provided antioxidant activity. During sprinting, there is an accumulation of reactive oxygen and nitrogen species which may be involved in fatigue development [33]. In the present study, it is likely that the antioxidant effect by the intake of the blackcurrant extract neutralised the reactive and nitrogen species’ ability to depolarise the sarcolemma, reduce the amount of calcium released from the sarcoplasmic reticulum, decrease calcium sensitivity of the thin filament and affect the myosin’s ability to bind to an actin filament [34]. However, the present study showed that the intake of New Zealand blackcurrant extract seemed to be more effective in trained football players during the running based anaerobic sprint test. This may be surprising as we cannot exclude that antioxidant ability was already higher in the trained football players due to higher training status. In addition, it may thus also be the case that the potential anti-oxidant effect of blackcurrant was not contributing to the observed sprint performance in trained youth football players in the present study.

Intake of New Zealand blackcurrant enhanced high-intensity treadmill running in a laboratory protocol that correlated with the YoYo IR2 test [14]. In addition, we observed that there was reduced slowing of the 20-m running sprint during the later stage of the Loughborough Intermittent Shuttle Test (i.e., LIST) [16]. Taken together, the observations in the present study during the RAST and our observations in previous ‘football’ specific tests, seem to suggest that the running ability of football players may benefit from the intake of New Zealand blackcurrant extract. However, due to the variety in intensity of running, in addition to the complexity of competitive football with non-running activities, we do not know whether intake of New Zealand blackcurrant has application for actual football performance.

Several limitations of the present study need to be mentioned. First, the repeated sprinting over a distance of 35 m and recovery time of 10 sec is not a consistent part of football matches at elite level [35]. Second, the present study did not control for dietary intake. Previous studies have restricted polyphenol intake [36,37], and such dietary control could have contributed to the observations of the study. In addition, the dietary intake for the second experimental visit was not quantified. Third, the application of a sprint threshold criterion to avoid pacing, did still result in some participants running faster in the sixth sprint compared to the fifth sprint. This could be explained by the central governor model which has suggested than an end spurt occurs with an individual having the ability to increase their speed for the final 10% of an exercise bout regardless of their previous level of exertion [38] or by the rating of perceived exertion template for pacing [39]. Fourth, the testing of both groups occurred on a different artificial surface which may have affected somewhat the absolute sprint times.

In summary, 7-day intake of New Zealand blackcurrant extract was associated with reduced fatigue during the running anaerobic sprint test. New Zealand blackcurrant also attenuated the increase in sprint times in trained youth football players. Our observations were likely caused by the anthocyanin composition of the New Zealand blackcurrant which has been shown to improve blood flow via increased nitric oxide and neutralised produced reactive oxygen species. These findings may have implications for nutritional strategies used by trained athletes involved in sports that consist of repeated maximal sprints.

## 5. Conclusions

Seven-day intake of New Zealand blackcurrant extract seems to be more beneficial for the ability to perform repeated sprints in trained football players. For football players, it seems that those with the ability to compete at a high level benefit more from the ergogenic effects of New Zealand blackcurrant. These findings may have implications for nutritional strategies used by trained athletes in sports that involve repeated maximal sprints.

## Figures and Tables

**Table 1:** Participant characteristics.

Parameter	Trained Youth Football Players (*n* = 9)	Recreationally Active Football Players (*n* = 15)
Age (years)	17 ± 0	20 ± 1
Height (cm)	178 ± 8	174 ± 19
Body mass (kg)	69 ± 9	80 ± 13
Body fat (%)	12.1 ± 2.1	15.9 ± 2.9
Sum of skinfolds (mm)	28 ± 5	40 ± 10
$\dot{V}$O_2max_ (mL·kg·min^−1^)	45 ± 5	44 ± 5
HR_max_ (beats·min^−1^)	200 ± 4	185 ± 10
Total MET (min·week^−1^)	12,393 ± 5375	7187 ± 4441
Anthocyanin intake (mg·day^−1^)	19 ± 23	11 ± 14

$\dot{V}$O_2max_, predicted maximum oxygen uptake; HR, heart rate; MET, metabolic equivalent. Data reported as mean ± SD.

**Table 2:** Sprint time and rating of perceived exertion (RPE) for each sprint during the running anaerobic sprint test (i.e., 6 × 35-m sprints) for trained youth and recreationally active football players.

Parameter	One	Two	Three	Four	Five	Six
Sprint time (s)						
Placebo	5.26 ± 0.32	5.38 ± 0.30 *	5.59 ± 0.29 *^,$^	5.68 ± 0.36 *^,$^	5.82 ± 0.42 *^,$^	5.77 ± 0.37 *^,$,#^
NZBC extract	5.25 ± 0.33	5.36 ± 0.30 *	5.51 ± 0.36 *^,$^	5.62 ± 0.42 *^,$,#^	5.73 ± 0.43 *^,$,#,£^	5.69 ± 0.40 *^,$,#^
RPE						
Placebo	8 ± 3	9 ± 3 *	11±3 *^,$^	13 ± 3 *^,$,#^	15 ± 3 *^,$,#,£^	16 ± 3 *^,$,#,£,@^
NZBC extract	7 ± 2	9 ± 2 *	11 ± 2 *^,$^	13 ± 2 *^,$,#^	15 ± 3 *^,$,#,£^	16 ± 3 *^,$,#,£,@^

Data reported as mean ± SD from 24 participants. * Difference with first sprint; ^$^ difference with second sprint; ^#^ difference with third sprint; ^£^ difference with fourth sprint; ^@^ difference with fifth sprint (*p* < 0.05). NZBC = New Zealand blackcurrant extract; RPE = rating of perceived exertion.
